# Oscillatory visual mechanisms revealed by random temporal sampling

**DOI:** 10.1038/s41598-021-00685-w

**Published:** 2021-10-29

**Authors:** Martin Arguin, Roxanne Ferrandez, Justine Massé

**Affiliations:** 1 Centre interdisciplinaire de recherche sur le cerveau et l’apprentissage (CIRCA), Département de psychologie, Université de Montréal, Montreal, Canada; 2 Centre de recherche, Institut Universitaire de Gériatrie de Montréal, Montreal, Canada; 3 Département de psychologie, Université de Montréal, Succ. Centre-ville, C.P. 6128, Montréal, QC H3C 3J7 Canada

**Keywords:** Neuroscience, Psychology

## Abstract

It is increasingly apparent that functionally significant neural activity is oscillatory in nature. Demonstrating the implications of this mode of operation for perceptual/cognitive function remains somewhat elusive. This report describes the technique of random temporal sampling for the investigation of visual oscillatory mechanisms. The technique is applied in visual recognition experiments using different stimulus classes (words, familiar objects, novel objects, and faces). Classification images reveal variations of perceptual effectiveness according to the temporal features of stimulus visibility. These classification images are also decomposed into their power and phase spectra. Stimulus classes lead to distinct outcomes and the power spectra of classification images are highly generalizable across individuals. Moreover, stimulus class can be reliably decoded from the power spectrum of individual classification images. These findings and other aspects of the results validate random temporal sampling as a promising new method to study oscillatory visual mechanisms.

## Introduction

There is growing evidence suggesting that functionally relevant neural communication takes the form of periodic synchronized firing by groups of neurons^1–4^. At a macroscopic level, this takes the form of oscillatory activity which can be recorded in humans by electroencephalography (EEG) or magnetoencephalography (MEG^5^).

In vision, investigation of the functional impact of brain oscillations has largely focused on attention. Thus, by recording brain activity with EEG or MEG or by interfering with it using transcranial magnetic stimulation (TMS), it has been determined that spatial attention operates at a rate of about 5–8 Hz and is driven by oscillatory brain activity in the high-theta/low-alpha frequency bands^6–11^.

As a general principle, if brain oscillations underlie faculties such as perception and cognition, then this should be manifest in behavioral performances. For instance, perceptual effectiveness should oscillate rapidly through time, in contrast to subjective experience which rather suggests a stable capacity. Empirical evidence in support of perceptual oscillations has been reported based on the periodicity of performance measures in humans carrying out various perceptual tasks^12–18^. The capacity of this approach to characterize putative oscillatory perceptual mechanisms remains limited, however, and a source for more potent and detailed evidence is clearly desirable. The new technique described below offers such advances which will be addressed in the discussion section.

Initially proposed by [^19^; see also^20^], we have enhanced the test and analysis procedures for the technique of random temporal sampling to make it a powerful tool to investigate oscillatory visual mechanisms. In addition to demonstrating temporal inhomogeneity in processing effectiveness, the experiments reported here also reveal crucial features of the oscillatory mechanisms involved. As shown below, some of these features are shared to a remarkable degree among observers and they vary substantially between stimulus conditions, such that they are diagnostic of the specific task participants perform.

**Figure 1:**
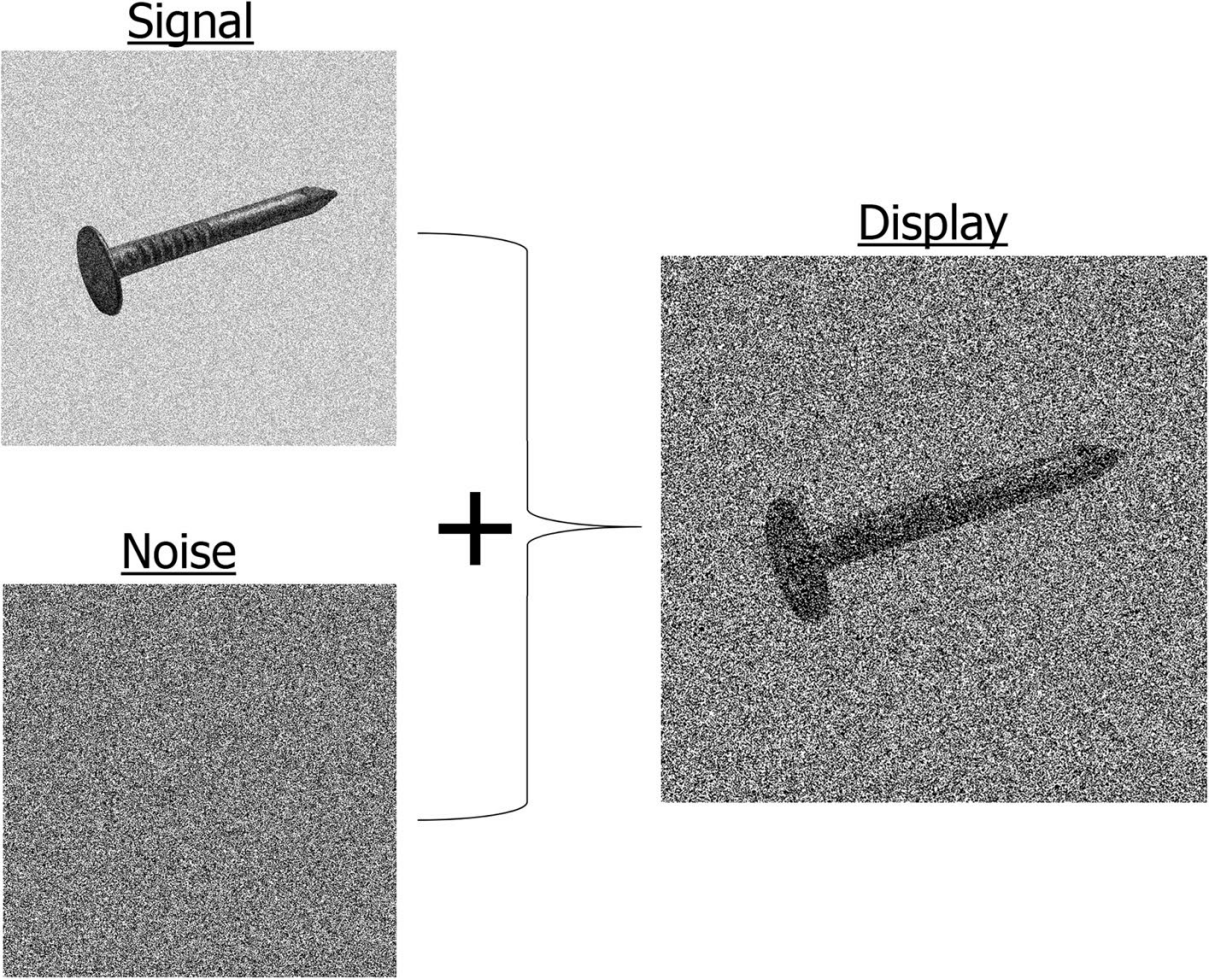
The stimuli displayed on every trial were made from the addition of two components, the signal and the noise. The ‘signal’ was made from an image of the target stimulus superimposed with a white noise field whose contrast was adjusted to maintain performance at about 50% correct (see “Methods”). The ‘noise’ was made from a separate white noise field with maximum contrast. New white noise fields were generated independently on each trial. The signal-to-noise ratio of the stimuli displayed was varied across their 200-ms exposure duration according to a new random function generated on each trial (see “Methods”).

Adult neurologically intact human observers carried out visual recognition tasks using words, familiar or novel objects, or faces. Throughout their exposure duration (200 ms), the stimuli were sampled by a manipulation of target visiblity (controlled by signal-to-noise ratio), which oscillated according to a random function. Specifically, the stimuli displayed were made from the linear combination of a target stimulus (the signal) and a white noise field (the noise; Fig. 1). The signal-to-noise ratio varied according to a complex pattern made from the integration of 5–55 Hz sine waves (in 5 Hz steps) with random amplitudes and phases, thus making the temporal sampling function. A new random temporal sampling function was generated on each trial thereby making them unpredictable.

**Figure 2:**
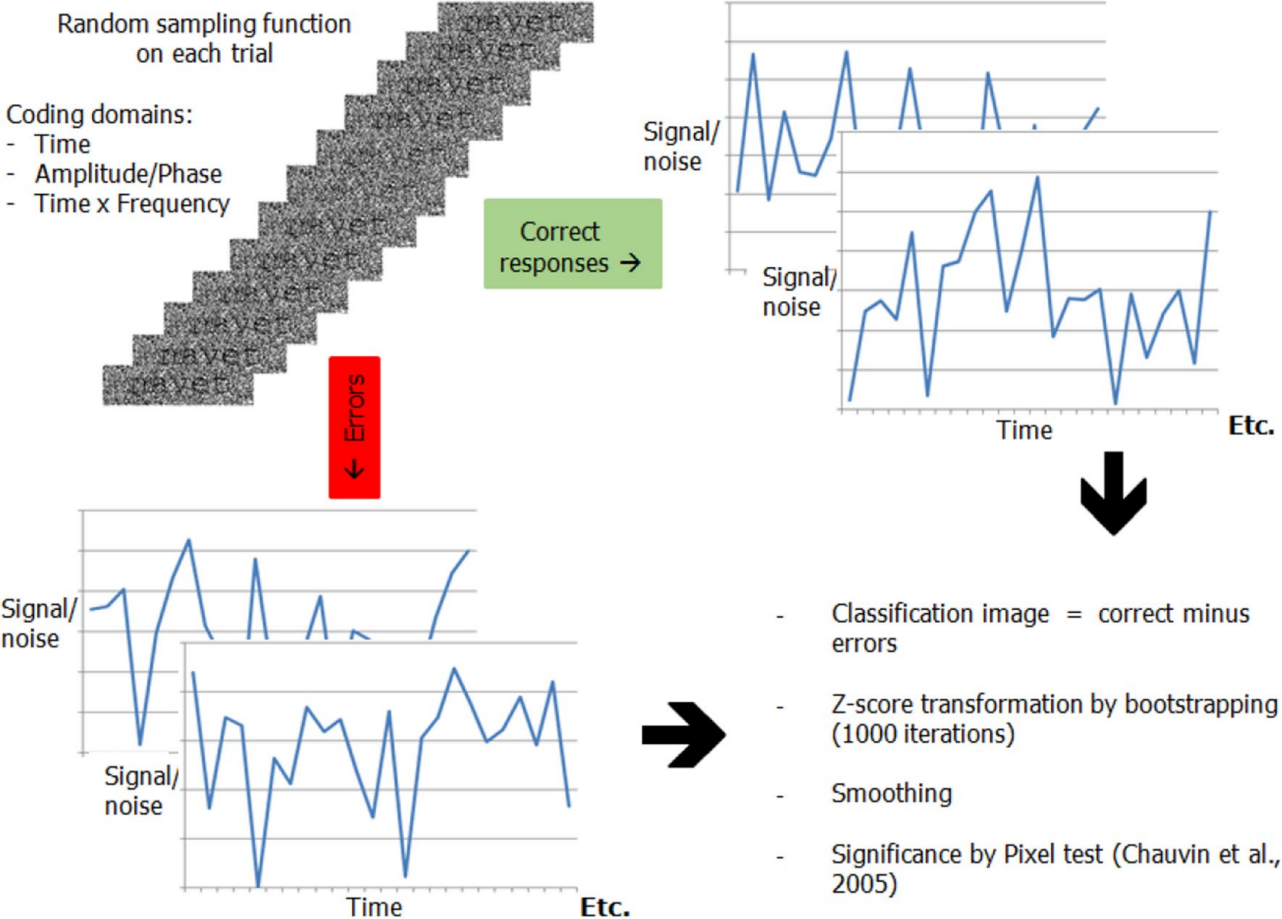
Illustration of the variability of the signal-to-noise ratio through time for the target word ‘navet’ (top left) and of the procedures applied to construct the classification images. The random temporal sampling functions generated on each trial were coded as either signal-to-noise ratio variations as a function of time since target onset (time domain), in terms of the amplitude and phase values of the 5–55 Hz (in 5-Hz steps) sinusoidal functions from which the temporal sampling functions were constructed (Fourier domain), or in terms of the instantaneous oscillation frequencies of the signal-to-noise ratio as a function of time since target onset (time–frequency domain). The signal-to-noise ratio vs. time sampling functions illustrated on the top right and bottom left of the figure are actual functions which were associated with either correct responses or errors in the word recognition task. The raw classification images were obtained from the weighted subtraction of the sums of the sampling functions (coded in the time, Fourier, or time–frequency domain) associated with errors from those associated with correct responses. They were then transformed into Z scores by bootstrapping, smoothing, and submitted to the Pixel test^22^.

Classification images^21^ of visual processing efficiency were then constructed by the weighted subtraction of the sampling functions on which participants made errors from those on which they produced a correct response (Fig. 2). For this operation, the sampling functions were either coded as signal-to-noise ratio variations through time (time domain), the power and phase values from which the sampling functions were constructed (Fourier domain), or the local temporal frequency content of these functions through time (time–frequency domain). Processing efficiency refers to the capacity of participants to use the stimulus information available to perform their task correctly.

After converting the processing efficiency metric of the individual raw classification images into Z scores by a bootstrapping operation, these classification images were averaged, smoothed and then submitted to the Pixel test[^22^; see “Methods” section for details] to determine the points that differed significantly from zero.

## Results

**Figure 3:**
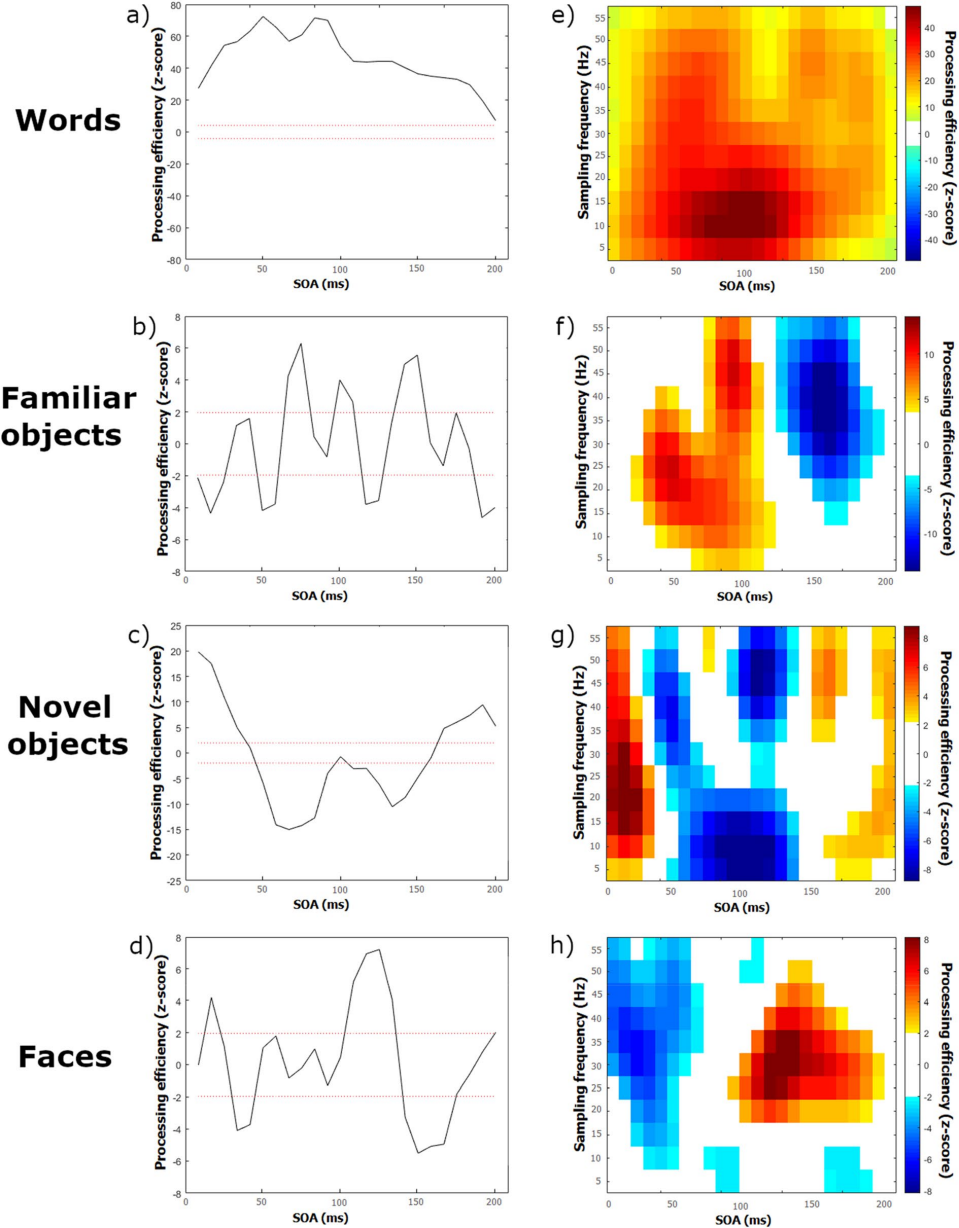
Classification images of encoding effectiveness (in Z scores) as a function of the time elapsed since target onset (stimulus onset asynchrony; SOA—panels **a**–**e**) and as a joint function of the frequency content of stimulus oscillations and SOA (panels **e**–**h**). Stimuli are: words (**a**,**e**), familiar objects (**b**,**f**), novel objects (**c**,**d**), and faces (**d**,**h**).

The classification images in Fig. 3 show variations of processing efficiency in the time and time–frequency domains for each stimulus class. The time domain classification images (Fig. 3a–d) illustrate processing efficiency as a function of the time elapsed since the beginning of target display (i.e. stimulus onset asynchrony; SOA). The red dotted horizontal lines indicate the significance thresholds for the Pixel test. Every portion of the classification images between those red lines does not differ significantly from zero, meaning that processing efficiency was effectively null, i.e. the target information available at these moments had no impact on performance. Portions of these classification images that are above the top red line indicate that participants were able to use the target information displayed at that particular time to benefit performance. In contrast, portions of the classification images that are below the bottom red line indicate that target information displayed at that time significantly interfered with performance. It is clear from these illustrations that processing efficiency varies substantially throughout the exposure duration of a visual target, whichever the stimulus class.

Processing efficiency according to a time–frequency representation of the temporal sampling functions is shown in Fig. 3e–h for each stimulus class. In these illustrations, points at which processing efficiency does not significantly differ from zero are in white. Warmer colours are associated with significant positive values of processing effectiveness (i.e. the target information available led to performance improvements) whereas cooler colours are associated with significant negative values (i.e. the target information displayed interfered with performance). It can easily be seen from these figures that visual processing efficiency varies not only according to SOA but that this effect interacts significantly with the temporal frequency content of the variations of target visibility through time. Again, these observations are verified for all the stimulus classes tested.

Classification images of static frequencies of stimulus oscillation based on the Fourier descriptors of the temporal profiles of stimulus sampling were also calculated. None of these classification images showed any significant difference from zero (see Supplementary Fig. S1), meaning that they fail to capture the temporal features of the target which impact processing efficiency. Here also, this finding characterizes each of the stimulus classes used in our experiments.

**Figure 4:**
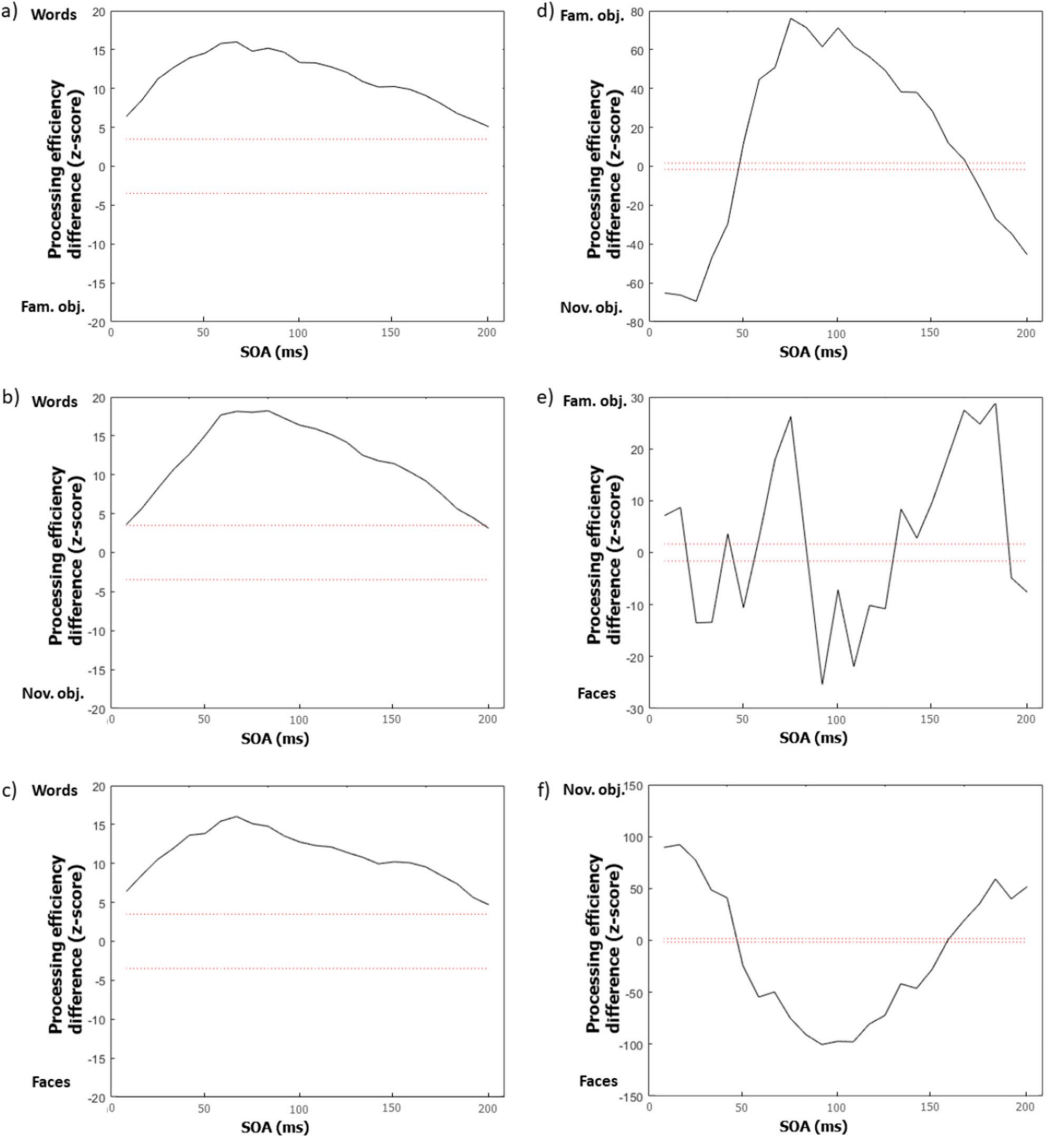
Contrast classification images in the time domain. Each graph expresses the differences in processing effectiveness (in Z scores) as a function the time elapsed since target onset (stimulus onset asynchrony; SOA) for a particular pair of tasks (defined by stimulus class). The ends of the ‘Processing efficiency difference’ scales are labelled by one of the stimulus classes being contrasted and difference values close to one end or the other end favour the corresponding stimulus class. All possible pairs of tasks have been contrasted: (**a**) words minus familiar objects; (**b**) words minus novel objects; (**c**) words minus faces; (**d**) familiar objects minus novel objects; (**e**) familiar objects minus faces; (**f**) novel objects minus faces.

**Figure 5:**
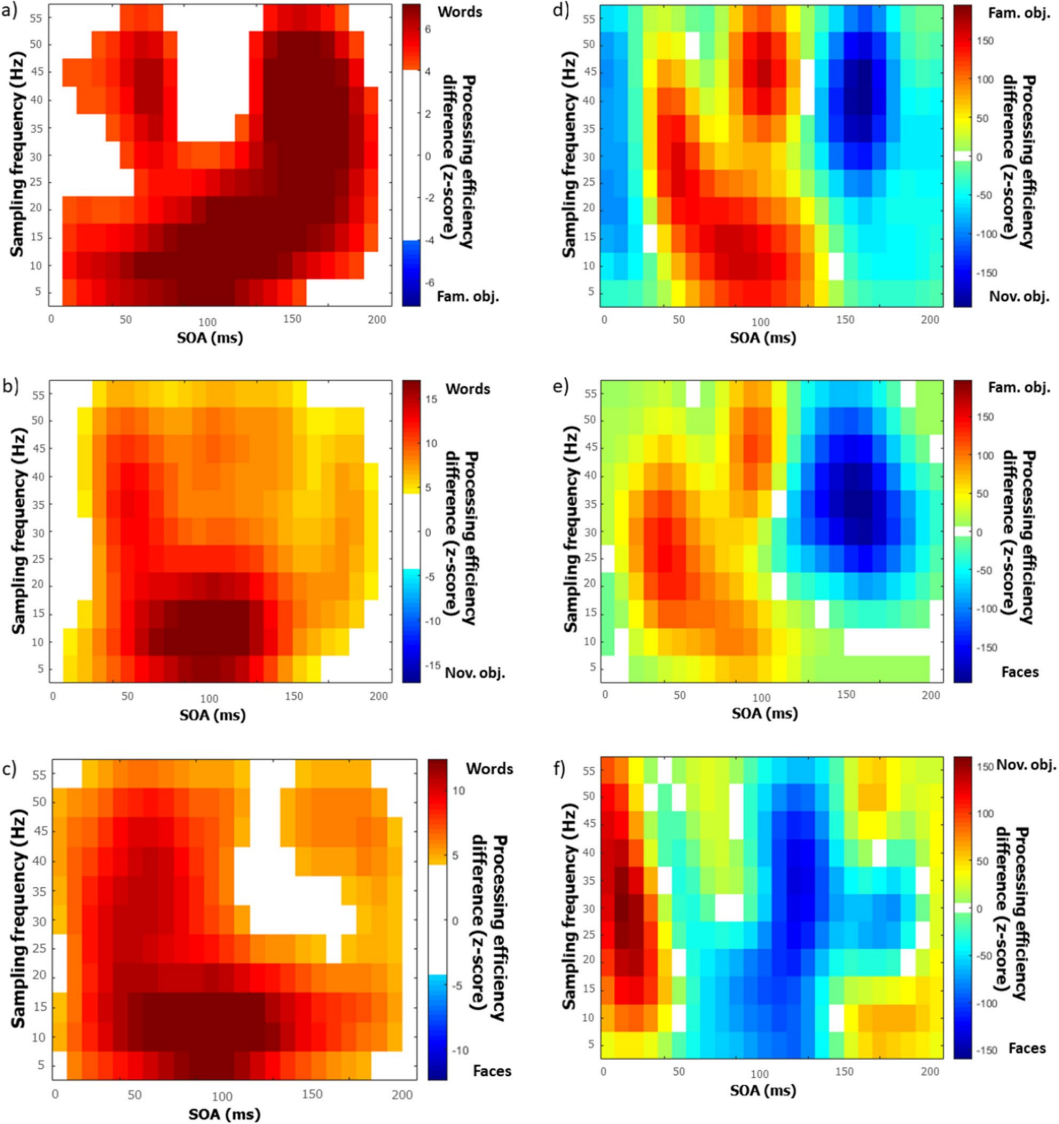
Contrast classification images in the time–frequency domain. Each graph expresses the differences in processing effectiveness (in Z scores) as a joint function of the frequency content of stimulus oscillations and SOA for a particular pair of tasks (defined by stimulus class). The ends of the ‘Processing efficiency difference’ scales are labelled by one of the stimulus classes being contrasted and difference values close to one end or the other end favour the corresponding stimulus class. All possible pairs of tasks have been contrasted: (**a**) words minus familiar objects; (**b**) words minus novel objects; (**c**) words minus faces; (**d**) familiar objects minus novel objects; (**e**) familiar objects minus faces; (**f**) novel objects minus faces.

Another important feature of the results that is apparent from the inspection of Fig. 3 is that the classification images, whether in the time or time–frequency domain, are very different according to stimulus class. This observation suggests that the temporal features that determine perceptual encoding are affected by the particular demands of the task that needs to be performed. This impression is confirmed by the contrast classification images displayed in Figs. 4 and 5. These were constructed by subtracting the classification image obtained with one stimulus class from that of another, with transformation into Z scores by bootstrapping, followed by smoothing, and the application of the Pixel test to determine where differences were significantly different from zero. Illustration conventions are as in Fig. 3.

One important issue we wanted to examine was the degree to which the classification images obtained from the random temporal sampling technique generalized across individuals. In other words, to what degree can the classification image obtained from one participant predict that from another? This question is important not only to determine how representative the present results are of the general population, but also to index the degree to which the mechanisms captured by the classification images shown in Fig. 3 are fundamental, or essential, for the visual system. To address this question, we calculated the agreement between individual classification images using the intraclass correlation coefficient (ICC^23^). The theoretical upper bound for the ICC is 1 and it has no lower bound. The mean ICC across stimulus classes is very low for both the time (mean ICC = − 0.90) and time–frequency (mean ICC = 0.07) classification images. Detailed results for each stimulus class as well as confidence intervals are shown in Supplementary Table S1.

Classification images however, do contain generalizable information which may be revealed once properly extracted. Thus, Fourier analyses were carried out on individual time domain classification images as well as separately for each stimulus sampling frequency (5–55 Hz in 5-Hz steps) of individual time–frequency classification images. They were thus decomposed into their power and phase spectra, which were then submitted to an assessment of between-subject agreement. This agreement was reasonably high for the phase spectra of the time domain classification images (mean ICC = 0.66 across stimulus classes) but very low for the time–frequency classification images (mean ICC = 0.06 across stimulus classes). In contrast, the power spectra of classification images were remarkably stable across participants for both types of classification images and all four stimulus classes (mean ICC across stimulus classes = 0.96 for the time domain; 0.98 for the time–frequency domain). The ICCs for the phase and power spectra of each type of classification image and for each stimulus class, along with their confidence intervals are shown in Supplementary Table S2.

The phase and power spectra of classification images were examined further with respect to their capacity of offering a pattern that was distinct according to the particular stimulus class participants have to recognize. An ANOVA on the phase spectra of time-domain classification images with frequency in classification image and stimulus class as factors showed no significant interaction (F(36, 480) = 1.7; *ns*). This means that the phase spectra of classification images did not differ significantly as a function of the stimulus class participants had to process. Similarly, the phase spectra of time–frequency classification images failed to reveal a task-specific pattern. Thus, the ANOVA with frequency in classification image × sampling frequency × stimulus class showed no significant interaction involving the latter factor (sampling frequency × class: F(30, 400) = 1.11; *ns*; classification image frequency × class: F(36, 480) < 1; 3-way interaction: F(360, 4800) < 1).

In contrast to the phase spectra, the power spectra of classification images offered distinct patterns depending on the stimulus class participants were required to recognize. Thus, the interaction of classification image frequency × stimulus class for the power spectra of the time domain classification images was highly significant (F(36, 480) = 8.3; *p* < 0.001). In the same vein, the 3-way interaction of sampling frequency × classification image frequency × stimulus class was significant for the power spectra of time–frequency classification images (F(360, 4800) = 1.3; *p* < 0.005).

**Figure 6:**
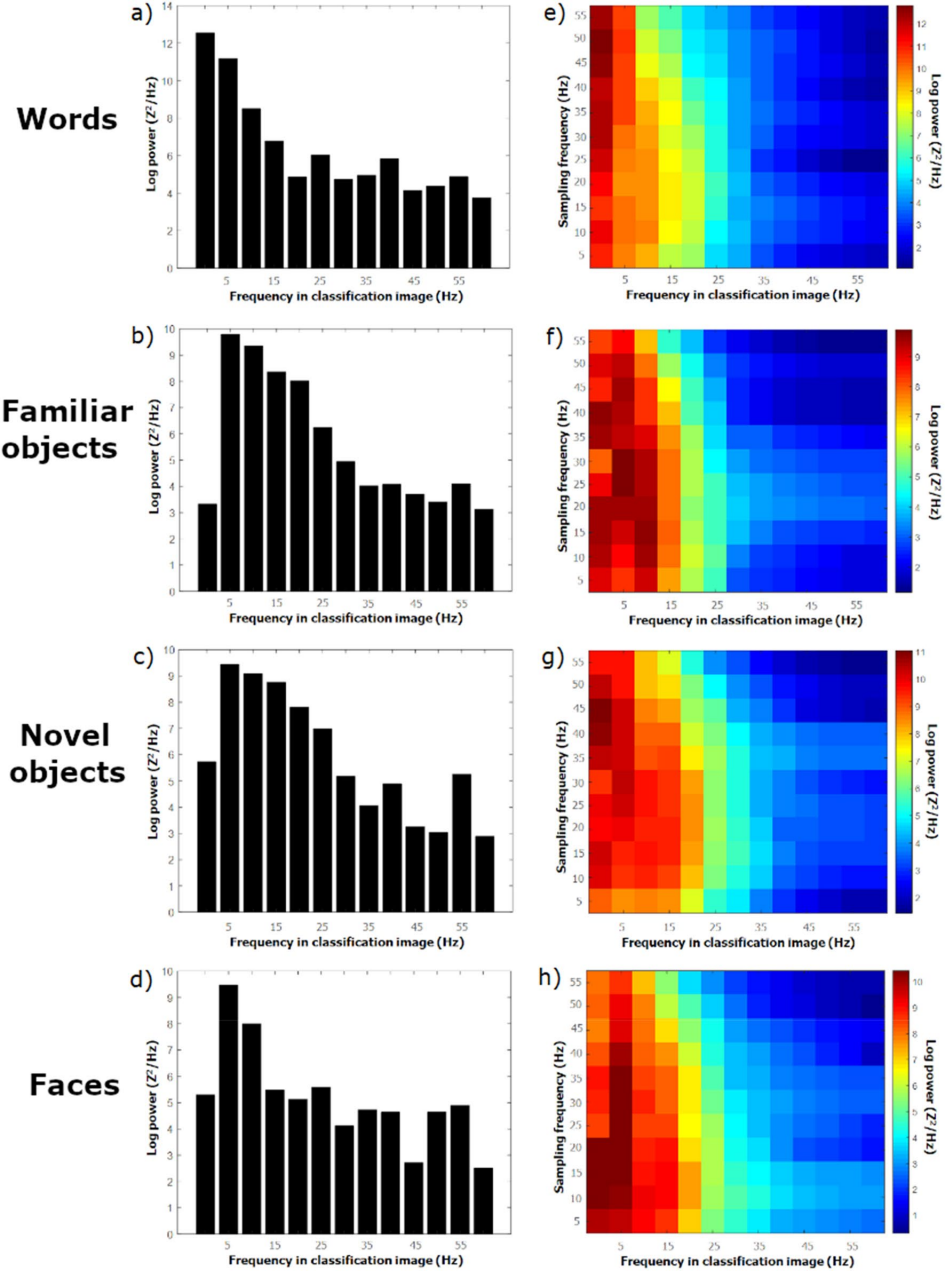
Power spectra of the classification images of encoding effectiveness in the time domain (panels **a**–**e**) and in the time–frequency domain (panels **e**–**h**). Stimuli are: words (**a**,**e**), familiar objects (**b**,**f**), novel objects (**c**,**d**), and faces (**d**,**h**).

The high between-subject agreement of the power spectra of individual classification images (shown in Fig. 6) along with their distinct patterns according to stimulus class suggests that this data may comprise signature properties that are diagnostic of the particular class of stimuli participants are required to recognize. This was determined with support vector machines (SVMs^24^) which were trained to map the power spectra of the classification images of individual participants to stimulus class with a leave-one out validation procedure. Out of four possibilities, stimulus class was predicted with 75% accuracy from the power spectra of the time domain classification images and with 52% accuracy using the power spectra of the time–frequency classification images. These correct classification rates are both well above chance (time domain: χ^2^ (9) = 68.9; p < 0.001; time–frequency domain: χ^2^(9) = 31.0; p < 0.001).

## Discussion

Four visual recognition experiments using the technique of random temporal sampling were carried out in normal adult observers with different stimulus classes. The classification images constructed from the relation between response accuracy and temporal features of stimulus sampling demonstrate that visual processing efficiency varies markedly throughout stimulus exposure in conjunction with stimulus oscillation frequencies (Fig. 3). These features falsify subjective impressions of the stability of visual capacity through time. They rather support the hypothesis that the neural systems underlying visual processing are oscillatory.

Somewhat surprisingly, the present results indicate that temporal and time–frequency domain classification images are quite variable across individuals and thus that their means may not constitute an ideal representation of the perceptual mechanisms studied. Indeed, the index of between-subject agreement was very low for both the time and time–frequency classification images.

A decomposition of these classification images into their power and phase components however, yields a more substantial picture. It demonstrates strikingly consistent power spectra among participants tested with the same stimulus class, with agreement indexes very close to the theoretical maximum of 1. In contrast, between-subject agreement is much lower for the phase spectra of classification images. This means that while their power spectra are nearly identical, the time and time–frequency classification images vary across participants because of mismatching phase spectra. These results indicate that different observers engage to the same degree a number of oscillatory mechanisms which are necessary to perform a given task. However, the temporal relations among these oscillations as well as with stimulus events is what varies substantially across observers. It is the latter which is responsible for the poor between-subject consistency of individual classification images.

In addition to excellent between-subject agreement, the power spectra of time and time–frequency classification images are rather unique for each task. Thus, their patterns differ significantly as a function of stimulus class. Taken together, these two features of the power spectra of classification images make it possible to use them to determine the stimulus class they originate from with significant accuracy. This observation strongly reinforces the idea that random temporal sampling taps fundamental visual mechanisms that are largely shared among neurologically intact adults.

### Differences among stimulus classes

The capacity of time and time–frequency domain classification images (Figs. 4, 5) as well as of their power spectra to discriminate reliably between stimulus classes indicates that the oscillatory mechanisms revealed are different or are engaged differently across experiments. This observation is congruent with the existence of specialized cortical mechanisms for visual word, object, and face recognition^25^. In turn, since functional specialization for stimulus classes only emerges at relatively high levels in the cortical hierarchy, this might suggest that random temporal sampling may tap neural mechanisms that are several synapses away from receptors.

A note of caution is required, however. Apart from stimulus class, there were several other differences between the experiments reported (e.g. stimulus sizes, brightness, background intensity, item familiarity, etc.) which are likely to have affected their outcomes. Thus, the present findings should be considered suboptimal for the purpose of characterizing the different oscillatory visual mechanisms involved in the recognition of the stimulus classes used. Our central purpose here is instead to demonstrate that the results obtained with random temporal sampling are highly sensitive to the changes in putative visual oscillatory mechanisms that are brought about by changing the observer’s perceptual experience and/or task. This is clearly the case considering the significant differences between the classification images obtained with different types of stimulus materials (Figs. 4, 5). Strong support for this conclusion is also provided by the successful classification of the stimulus class participants had to recognize on the basis of the power spectra of their time or time–frequency domain classification images.

Another interesting aspect of the present results that may be noticed from inspection of Figs. 3, 4, 5 is that processing efficiency (i.e. z-scores) is substantially greater in the task of word recognition than for other stimulus classes. For instance, the processing efficiency peaks for words in the time and time–frequency classification images are of 72.5 and 49.9 respectively, whereas they are of 6.3 and 11.9 for familiar objects, 19.7 and 9.6 for novel objects, and 7.2 and 9.2 for faces. This is an observation we had not anticipated. We speculate that this may relate to differences among stimulus classes with respect to the amount of information (in the classic sense of “reduction of uncertainty”^26^) that is required for their accurate recognition.

Indeed, word recognition is known to largely proceed through the identification of its constituent letters^27^, of which only 26 possibilities exist in French. This means that there is a finite number of five-letter strings that may possibly exist (i.e. 26^5^ items) and this number is largely reduced if we only consider items made of allowable letter sequences (within pairs or triplets) in the language. A further constraint which applies here is that the letter sequences presented must constitute a French word, which are rather few (2048 orthographically distinct five-letter words in the BRULEX database^28^, which was used here for stimulus selection). These different factors constitute a priori probabilities that human vision most probably applies for word recognition^29^, thereby reducing the quantity of information that is needed to identify a word compared to stimulus classes such as familiar objects or faces, for which no such constraints apply. For our novel object recognition task, the number of object identities was quite low (n = 6) but it would seem reasonable to argue that the amount of information required for their recognition was higher than for words given their initial unfamiliarity and the use of varied viewpoints.

Then, under the assumption that the rate of information transmission by the visual system is limited, the brief instants of stimulus visibility that were available allowed the encoding of visual information which may have had a greater potential to lead to correct target recognition in the case of words compared to other stimulus classes. Expressed in another way, the account proposed here for the greater processing efficiency for words than for familiar or novel objects, or faces, is that the scores reflecting processing efficiency would be inversely related to the amount of information that is required to accomplish the task correctly. We suggest it would be of interest for future research to investigate this idea.

### Static versus transient oscillations

A noteworthy finding from the present study is that classification images of processing effectiveness as a function of the static temporal frequencies present in the sampling functions (i.e. Fourier domain classification images) revealed no significant effect (see Supplementary Fig. S1). This finding demonstrates the incapacity of static temporal frequencies of stimulus visibility oscillations to modulate visual recognition performance. This contrasts with the time–frequency classification images (Fig. 3e–h), which show the large impact of transient stimulus oscillations. These observations appear relevant to the issue of whether the neural oscillations that may be captured in humans by EEG/MEG reflect sustained or transient-burst events^30^. Specifically, the ineffectiveness of the static frequencies constituting the temporal sampling functions to modulate performance seems to suggest that the temporal sampling method used here would only impact transient-burst events while producing no evidence for sustained oscillations.

We must point out however, that the power spectra extracted by Fourier analysis from the individual time and time–frequency classification images do reflect sustained oscillations. As demonstrated above, these patterns of sustained oscillations are extremely similar across individuals and they can effectively serve to determine the stimulus class participants had to recognize.

This leads us to suggest that the technique of random temporal sampling may effectively tap both transient and sustained oscillatory visual mechanisms and that both provide a significant contribution to accurate performance.

### Temporal sampling vs. behavioral periodicity paradigms

The above conclusion that sustained oscillatory mechanisms are important for visual performance is congruent with the previous findings obtained from the behavioral periodicity paradigms discussed in the Introduction. Indeed, the demonstration of the periodicity of a particular phenomenon using these methods requires an oscillation that is sustained for some duration. It is unclear whether evidence for transient oscillations can be provided through the measurement of behavioral periodicity and, to our knowledge, no such demonstration is presently available.

Across tasks, the static oscillation frequencies—i.e. those extracted from individual classification images by Fourier analysis—that seem the most engaged range from 5 Hz to about 25 Hz, as can be seen from the frequencies in classification images that carry the most power (Fig. 6). However, substantial energy peaks can also be observed in the 35–55 Hz range, particularly in the novel object and face recognition tasks (Fig. 6c,d,g,h). The range of oscillatory frequencies revealed in the present study is substantially greater than those reported in previous studies using a method based on the periodicity of performance indicators[^12–18^; see “Introduction”]. Most significantly, the highest relevant frequencies documented here seem to reach as high as 55 Hz whereas behavioral periodicity studies have so far detected oscillation frequencies up to a maximum of about 20 Hz^18^. The sensitivity of the random temporal sampling technique to high frequency functional oscillations is clearly advantageous relative to alternative methods. This advantage is especially obvious when one considers the fundamental importance of neural oscillations in the gamma frequency band (30 Hz and above) for a vast range of cognitive and perceptual functions^1–3^, including visual–spatial attention^31^.

Two other practical considerations that also favour the temporal sampling technique over behavioural periodicity methods are the numbers of trials required to obtain proper data and the level of complexity of the experimental procedures they require. With regards to numbers of trials, periodicity methods may be somewhat prohibitive (several thousands of trials) in comparison to the 1200 trials per participant used here. Moreover, the procedure required to measure periodicity in perceptual performances may be quite complex whereas it is very straightforward with temporal sampling once the method involved in producing stimulus oscillations is in place.

### Synchronization with stimulus events

What appears critical for the ability of random temporal sampling to deliver relevant observations is for perceptual mechanisms to synchronize precisely with stimulus events. Indeed, without synchrony, the timing relations between the state of the visual system and stimulus events would be random. This would prevent classification images, which are based on large collections of trials, from showing variations of processing efficiency according to the timing of stimulus events since this relation would be entirely blurred. To prevent such problems, great care was taken in designing the experiments to present preparatory signals that would permit participants to precisely anticipate the moment of target onset and to maximize their level of alertness at that instant^32,33^. Also, the features of the auditory tone preceding this event were selected based on a prior demonstration of their capacity to reset neural oscillations in the visual system^34^. Future studies will be required to assess the actual value of these preparatory signals.

### Truly oscillatory mechanisms?

In the present study, we have used variations in visual processing effectiveness according to the temporal features of stimulus sampling as a way to probe putative oscillatory mechanisms in the human visual system. One question that may be asked with respect to the evidence obtained is whether it truly reflects visual mechanisms that are oscillatory. Indeed, there is an ongoing debate in the literature as to whether the oscillatory neural activity resulting from exposure to rhythmic stimuli reflects the response of an actual oscillator (i.e. “a system capable of generating sustained rhythmic behaviour by itself”^35^ p. 2) or simply neural entrainment by the periodicity of the stimulus; that is, a steady-state response^35–37^.

There are three aspects to the observations reported here that are relevant to the issue and which militate for an interpretation of our results in terms of visual mechanisms that are actual oscillators.

One is that the Fourier domain classification images showed that the stable rhythmic features of stimulus sampling had no significant impact on behavioral performance (Supplementary Fig. S1). Such an impact is precisely what would have been predicted by the hypothesis of neural entrainment.

Second, the temporal frequency content of stimulus sampling mainly had transient effects on behavioural performance, as shown by the time–frequency classification images (Fig. 3e–h). This observation is incompatible with the notion of neural entrainment. Take for instance the response to 50 Hz stimulus oscillations in Fig. 3f, which is the time–frequency classification image for familiar objects. At some SOAs, these oscillations have no impact on performance but at others, they are associated to either increased or decreased processing effectiveness. We see no way in which such a pattern could be accounted by neural entrainment.

Third, and finally, the power spectra of the time and time–frequency domain classification images of individual participants, which probably constitute the most potent observations from the present study, are not directly related to stimulus oscillations. They rather reflect intrinsic properties of the visual processing system that drive the variations of processing effectiveness through time that occur in individual time and time–frequency domain classification images.

### Note added in proof:

We have recently completed analyses for a new experiment involving one condition that was identical to the word recognition task reported here. These new results closely replicate those reported here in every respect.

## Methods

### Participants

All 44 participants, 29 women and 15 men, were French speaking neurologically intact undergraduate students at Université de Montréal aged between 19 and 35 years old. All add normal or corrected vision. Eight participants took part in the word recognition experiment, whereas distinct groups of 12 participants took part in each of the others, which were conducted later.

The recruitment of participants was initiated only after approval of the studies by the relevant ethics committee of the Université de Montréal (Comité d’éthique de la recherche en éducation et psychologie). All participants gave their informed consent to participate and all procedures were carried out in accordance with the relevant guidelines and regulations.

### Materials and stimuli

All experiments were run on an HPZ230 computer equipped with an NVIDIA GeForce GTX970 videocard and an ASUS VG248QR HD monitor with maximum luminance of 200 cd/m^2^ and a 120 Hz refresh rate. All stimuli were achromatic and all manipulations of intensity were linear. Experiments were programmed in Matlab [Mathworks Inc.] and made the use of the Psychophysics toolbox^38^. Observation distance was 57 cm, which was stabilized by having participants rest their head on a chin rest.

For the word recognition experiment, stimuli were 600 five-letter French common words with a mean frequency of 157 per million^28^. Words were printed in Tahoma (x-height of 0.76 deg) in black letters over a grey (half of maximum intensity) background.

For the familiar object recognition experiment, stimuli were greyscale photographs (from the Bank of Standardized Stimuli; BOSS^39^) of 300 objects shown in front of a white background. The items were selected based on the published performance statistics for BOSS images so that it would be easy for participants to recognize them and find their name. The maximum horizontal extent of the stimuli was 17.4 deg of visual angle and their maximal vertical extent was 17.1 deg.

**Figure 7:**
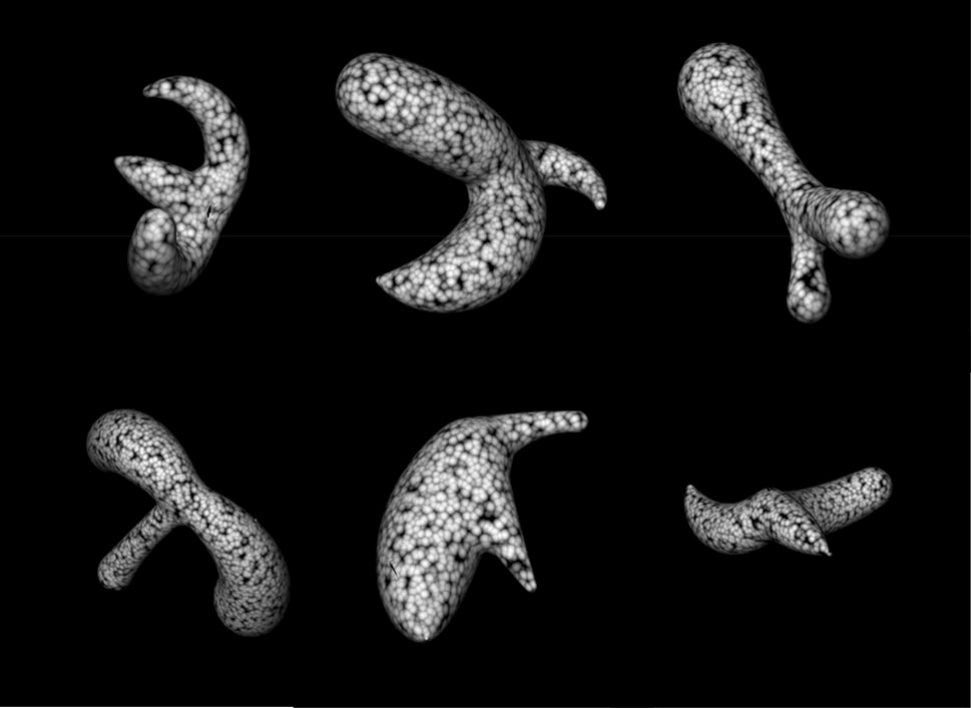
Illustration of a subset of the stimuli used in the novel object recognition task. The figure shows one instance of each object of the set. In the experiment, each object could be shown from one of four different viewpoints.

The stimuli used for the novel object recognition task were simple randomly generated 3-D shapes with two interconnected medial axes around each of which a cylindrical shape of variable diameter was built (Fig. 7; this stimulus class has been used previously by^40^. The stimuli displayed in the experiment were the 2D renderings of these items, lighted from above, covered in a rich achromatic texture and presented in one of four viewpoints, all of which revealed the major features characterizing the shape of the object. Items were shown over a black background and their maximum horizontal and spatial extent was of 9.5 deg.

One hundred and thirty greyscale photographs of famous actors and actresses from the face bank of^41^ were used for the face recognition experiment. The items selected were those which appeared as most likely to be recognized by participants. Each face appeared in frontal view and its expression was either neutral or happy. These photographs were displayed over a white background and their horizontal and vertical spatial extent was of 7.0 deg.

In all experiments, the duration of the target stimulus was of 200 ms. Target stimuli were made from the linear summation of two components, the signal and the noise (Fig. 1). The signal was made from the image of the target, as described above, overlaid by a white noise field with a contrast level that was adjusted on a trial by trial basis, as described below, in an attempt to maintain response accuracy to 50% correct. The noise was made from a second, independent white noise field of maximal contrast. Throughout the stimulus duration, the signal-to-noise ratio (SNR), that is the ratio of the weights attributed to the signal and the noise for the construction of the stimulus to be displayed, varied according to a random function constructed by the integration of sine waves with frequencies ranging between 5 and 55 Hz in steps of 5 Hz with random amplitudes and phases. A new, independent SNR function was generated on each trial. Examples of actual temporal sampling functions are illustrated in Fig. 2. The range of the SNR was normalized between 0 and 0.5 and its sum across the sequence of 24 image frames making up the 200 ms stimulus duration (at the refresh rate of 120 Hz) was matched across trials. The overall luminance and contrast of the stimuli were matched across image frames and across trials.

### Procedure

In all experiments, participants completed a total of 1200 trials over two test sessions of 600 trials each. In all cases, the participant’s task was to identify the target without time pressure. With word stimuli, participants had to read the word aloud. With familiar objects, they were required to identify the item using a name that was sufficiently specific to demonstrate accurate recognition. With novel objects, they had to indicate orally the digit assigned to it during the learning phase, which is described below. With famous faces, participants were asked to either give the name of the actor/actress, the name of his/her character in a particular movie or his/her specific role in that movie.

In the sequence of trials in the word, familiar object, or face recognition experiments, no item could be repeated before the complete stimulus set had been presented for the same number of trials. In the novel object recognition task, the order of presentation of items was random, with the constraint that each object be presented an equal number of times.

For the novel object recognition experiment, participants first had to be trained with the objects and the name they were assigned, which was a digit ranging from 1 to 6. This began with a familiarization phase during which participants were exposed to a sheet of paper on which the six objects were printed in all four viewpoints along with the digit associated with each object. Once the participant felt having studied the sheet sufficiently, the practice phase followed during which each of the possible 24 stimulus instances were presented eight times in a random trial sequence presenting one non-degraded item at a time until the participant named it. A correct response was followed by a 1000 Hz (50 dB pure tone) auditory feedback whereas errors were followed by a 300 Hz tone. Following each response, whether correct or not, the correct digit identity of the item was displayed on the screen for 500 ms. To move to the experimental phase, participants had to obtain at least 90% correct responses in a practice block.

In all experiments, the time course of each trial was as follows. A square white noise field of 18° to a side centred on the middle of the display monitor was first presented for 1250 ms. A white fixation cross was then added at the centre of the monitor for 250 ms, which then disappeared. This offset was followed 150 ms later by a 900 Hz–60 dB–14 ms pure tone announcing the onset of the target stimulus 100 ms later. The target stimulus was presented in the middle of the screen for a duration of 200 ms during which the SNR varied according to a random function, as described above. Following its offset, only the background white noise mask remained visible until the participant’s response. A 500 ms delay then followed prior to the beginning of the following trial.

Response accuracy on experimental trials was maintained to about 50% correct using a staircase procedure which automatically adjusted the contrast of the white noise superimposed on the target image as part of the “signal” portion of the stimulus to be displayed (see above). The available range of white noise contrast had 128 levels, the lowest producing a zero-contrast (i.e. null) mask and the highest being made of black and white (i.e. minimum vs. maximum luminance) elements. The first experimental block began with a white noise contrast exactly at the middle of the available range for the first 10 trials. Starting on trial 11, the average response accuracy for the 10 preceding trials was determined. If accuracy was exactly 50%, white noise contrast remained the same for the following trial. If accuracy was below 50%, white noise amplitude was reduced by one step (see below) whereas it was increased by one step if accuracy was above 50%. Initial step size was of 16 contrast levels and this value was halved every time the direction of adjustment was reversed, down to a minimum of 1. The state of the algorithm adjusting this white noise contrast was maintained across consecutive blocks of trials.

### Data analysis

Response accuracy was averaged across participants separately for each experiment. The average percentages of correct responses are; words: 60.5%; familiar objects: 49.2%; novel objects: 56.0%; and faces: 30.1%.

As indicated above, the contrast of the white noise mask that was part of the “signal” portion of the stimuli displayed was adjusted to maintain response accuracy to about 50% correct. The means of noise levels for each experiment are; words: 111.3; familiar objects: 119.3; novel objects: 119.9; and faces: 11.0, over a possible maximum of 128.

The main analyses pertained to the construction of classification images representing how response accuracy was affected by various temporal features of the sampling functions of the target stimuli (see Fig. 2). The temporal features which were analysed are: 1—SNR amplitude as a function of time (time domain) from target onset (or stimulus onset asynchrony; SOA), 2—the Fourier descriptors for these functions (Fourier domain) and, 3—time–frequency representations of these same functions (time–frequency domain). The Fourier descriptors are the amplitude and phase of the temporal frequencies (5–55 Hz in 5 Hz steps) used to build individual SNR time functions (see above). The time–frequency representations of the SNR functions on individual trials were calculated with a wavelet analysis using three-cycle complex Morlet wavelets varying in temporal frequency from 5 to 55 Hz in 5 Hz steps^42^. The number of cycles in the Morlet wavelet serving as the kernel in wavelet analyses was chosen to offer high precision in the time domain, which is the crucial issue we wanted to investigate. This leads to a sacrifice in the precision of measurements in the frequency domain, which implies sensitivity of the wavelet not just to its particular temporal frequency but to a range of frequencies around it.

For each mode of coding the SNR time functions, classification images were obtained for each individual participant. This was done by the weighted subtraction of the sums of the temporal features (i.e. SNR as a function of time, Fourier descriptors of these SNR functions, or time–frequency representation of SNR functions) of the sampling functions associated to errors from those associated to correct responses. These raw classification images were then transformed in Z scores by a bootstrapping operation whereby the sampling functions were randomly assigned to response accuracies while allowing for repetition, and from which classification images were constructed. The mean and standard deviation of 1000 such random classification images for an individual participant served as reference to transform the values from his/her raw classification image into Z scores.

Once transformed to a common scale, the individual classification images were averaged, smoothed, and then submitted to a two-way Pixel test^22^ with α = 0.05 to determine the points in classification images which differed significantly from zero. The Pixel test is derived from random field theory and has been applied for about the last 30 years for the analysis of brain imaging data. Its purpose is to establish the Z value that will serve as the significance criterion for a Z-scored image. Among others, factors that will affect the Pixel test is the spatial correlation inherent in the data set, the dimensionality of the latter, and the width of the filter used to smooth the data. In the case of the classification images reported here, the smoothing filter was Gaussian and had a full width at half maximum (FWHM) of 0.6 unit in the time domain and of 1.5 units in the time–frequency domain. Since we wanted to identify data points that were either significantly above or below zero, the Pixel test was two-way. Thus, to achieve an overall α of 0.05, the input given to the algorithm performing the Pixel test requested α = 0.025. The criterion Z score obtained was then used in its positive value to identify points that were significantly above 0 and in its negative value (i.e. Z_crit_ * − 1) to identify points significantly below 0.

Analyses of the agreement, or consistency of classification images across participants having taken part in the same experiment were carried out using the intraclass correlation coefficient (ICC^23^). The same procedure was followed to analyse the consistency among participants of the power and phase spectra of their classification images. The ICC assesses the similarity between participants of a group in their pattern of results across an array of measurements. The upper bound of the ICC is one and it has no lower limit. Supplementary Tables S1 and S2 report every ICC discussed in the present article, along with their 95% confidence intervals.

The analysis of raw individual classification images into their power and phase components was performed by one-dimensional fast Fourier transforms applied to either the time domain classification images or to Z score amplitude variations through time separately for each temporal frequency represented in time–frequency classification images (i.e. 5–55 Hz in 5-Hz steps).

Mixed-factor analyses of variance (ANOVAs) were conducted to examine whether the phase and power spectra of the time and time–frequency domain classification images showed different patterns according to stimulus class. Given the specificity of the question addressed by these analyses, only the relevant interactions are reported in “Results” section.

Stimulus class was decoded from the power spectra of individual time domain and time–frequency domain classification images using linear support vector machines (SVMs^24^) and a leave-one-out cross-validation procedure. Thus, the classification images of all but one participant were presented to the SVM for it to learn the mapping from classification images to stimulus class. Then, the classification image from the participant having been left out of the learning phase was presented to the SVM for it to determine which of the four stimulus classes had been presented. This process was repeated by leaving out a different participant on each iteration until it had iterated through all participants. Classification accuracy was determined from the percentage of iterations on which the SVM determined correctly the stimulus class processed by the left-out participant. Chi-square analyses were used to assess whether classification accuracy deviated significantly from chance.

## Supplementary Information

Supplementary Information.
