# Supplementary material for: Oscillatory visual mechanisms revealed by random temporal sampling
Source: Sci Rep. 2021 Oct 29;11:21309. doi: 10.1038/s41598-021-00685-w (PMC8556381; doi:10.1038/s41598-021-00685-w)
Supplement: Supplementary file 1 — Supplementary Information. [file 41598_2021_685_MOESM1_ESM.pdf]

Supplementary Information for “Oscillatory visual mechanisms revealed by random temporal sampling”, by Arguin, Ferrandez & Massé.

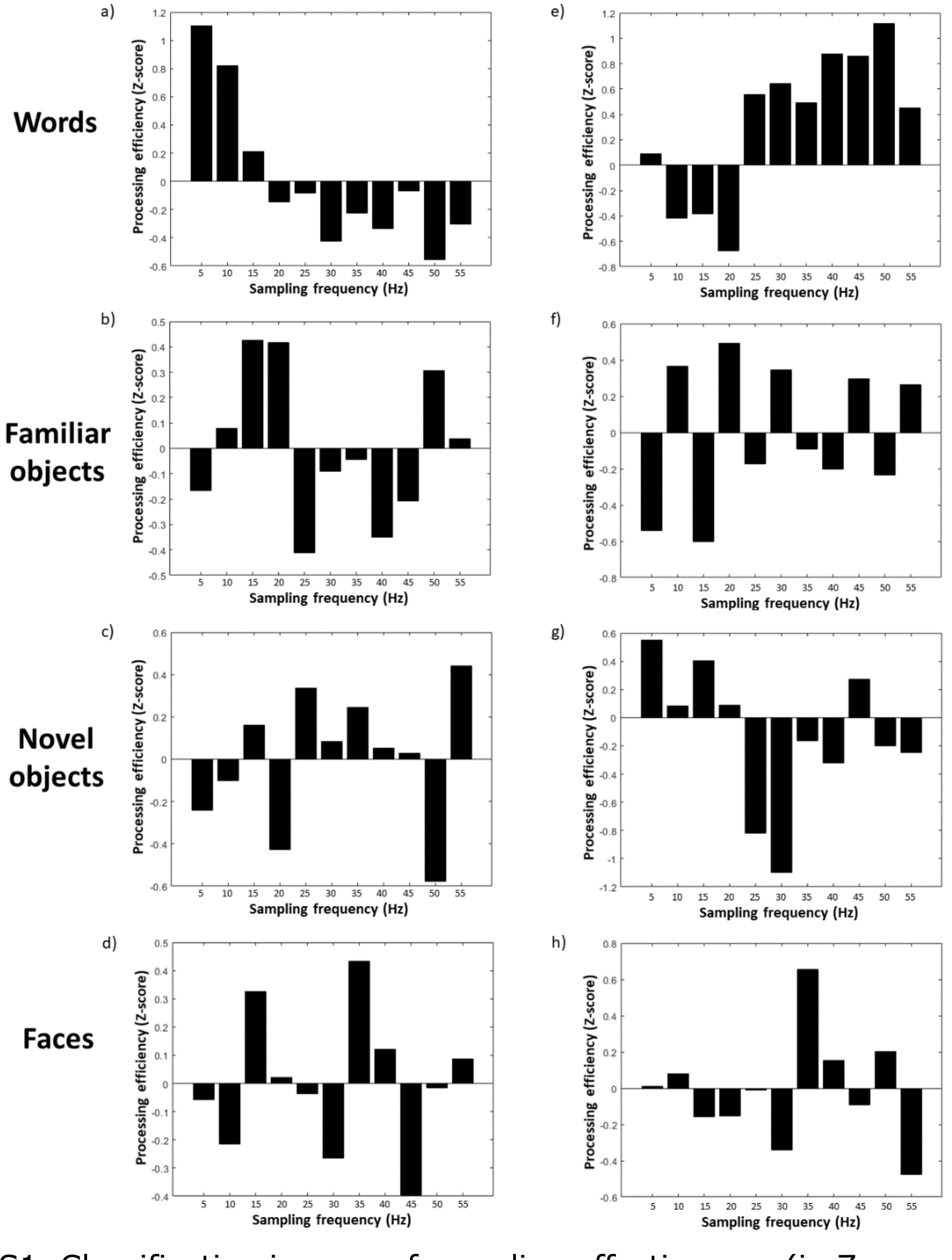

Fig. S1. Classification images of encoding effectiveness (in Z scores) as a function of the power spectrum (panels a-e) or the phase spectrum (panels e-h) of the temporal sampling function. Stimuli are: words (a and e), familiar objects (b and f), novel objects (c and d), and faces (d and h). None of the values illustrated in these graphs differs significantly from 0, based on the two-way Pixel test with α = .05.

Table S1

Intraclass correlation coefficients (i.e. ICC) for the time and time-frequency domain classification images for each stimulus class. The 'Lower limit' and 'Upper limit' associated with each ICC correspond to their 95% confidence intervals.

	**Time domain**			**Time-frequency domain**		
	ICC	Lower limit	Upper limit	ICC	Lower limit	Upper limit
**Words**	0.89	0.81	0.95	0.70	0.64	0.75
**Familiar objects**	-3.59	-6.85	-1.29	0.61	0.54	0.68
**Novel objects**	0.76	0.60	0.88	-0.08	-0.28	0.10
**Faces**	-1.65	-3.53	-0.32	-0.94	-1.30	-0.61

Table S2

Intraclass correlation coefficients (i.e. ICC) for phase spectra (a) and the power spectra (b) of the time and time-frequency domain classification images for each stimulus class. The 'Lower limit' and 'Upper limit' associated with each ICC correspond to their 95% confidence intervals.

a)

	**Time domain - Phase**			**Time-frequency domain - Phase**		
	ICC	Lower limit	Upper limit	ICC	Lower limit	Upper limit
**Words**	0.88	0.74	0.96	0.30	0.11	0.46
**Familiar objects**	0.57	0.12	0.85	-0.17	-0.47	0.10
**Novel objects**	0.70	0.40	0.89	0.24	0.04	0.41
**Faces**	0.49	-0.05	0.82	-0.12	-0.41	0.14

b)

	**Time domain - Power**			**Time-frequency domain - Power**		
	ICC	Lower limit	Upper limit	ICC	Lower limit	Upper limit
**Words**	0.96	0.91	0.99	0.97	0.97	0.98
**Familiar objects**	0.97	0.93	0.99	0.97	0.97	0.98
**Novel objects**	0.97	0.94	0.99	0.98	0.97	0.98
**Faces**	0.96	0.91	0.98	0.98	0.97	0.98
